# Combining Straw Mulch with Nitrogen Fertilizer Improves Soil and Plant Physio-Chemical Attributes, Physiology, and Yield of Maize in the Semi-Arid Region of China

**DOI:** 10.3390/plants12183308

**Published:** 2023-09-19

**Authors:** Kashif Akhtar, Weiyu Wang, Ivica Djalovic, P. V. Vara Prasad, Guangxin Ren, Noor ul Ain, Muhammad Riaz, Yongzhong Feng, Gaihe Yang, Ronghui Wen

**Affiliations:** 1 State Key Laboratory for Conservation and Utilization of Subtropical Agro-Bio-Resources, College of Life Science and Technology, Guangxi University, Nanning 530004, China; kashif@zju.edu.cn; 2 College of Agronomy, Northwest A & F University, Yangling, Xianyang 712100, China; wangweiyu0830@nwafu.edu.cn (W.W.);; 3 Institute of Field and Vegetable Crops, National Institute of the Republic of Serbia, Maxim Gorki 30, 21000 Novi Sad, Serbia; maizescience@yahoo.com; 4 Department of Agronomy, Kansas State University, Manhattan, KS 66506, USA; vara@ksu.edu; 5 Shenzhen Branch, Guangdong Laboratory for Lingnan Modern Agriculture, Genome Analysis Laboratory of the Ministry of Agriculture, Agricultural Genomics Institute at Shenzhen, Chinese Academy of Agricultural Sciences, Shenzhen 518120, China; noorulainali001@gmail.com; 6 Department of Environmental Sciences and Engineering, Government College University Faisalabad, Allama Iqbal Road, Faisalabad 38000, Pakistan; mr548@ymail.com

**Keywords:** straw mulching, photosynthesis, plant and soil health, water use efficiency

## Abstract

Mulching and nitrogen (N) fertilization are the main drivers for sustainable crop production. The sole use of nitrogen fertilizer threatened both the physiology and production of maize in rain-fed areas. Therefore, we proposed that wheat straw mulching with N fertilization would increase maize yield by improving soil fertility, physiology, and nitrogen use efficiency. A two-year field study evaluated the effects of CK (control), N (nitrogen application at 172 kg ha^−1^), HS (half wheat straw mulch, 2500 kg ha^−1^), HS+N (half wheat straw, 2500 kg ha^−1^ plus 172 kg N ha^−1^), FS (full wheat straw, 5000 kg ha^−1^), and FS+N (full wheat straw, 5000 kg ha^−1^ plus 172 kg N ha^−1^) on maize growth, physiology, and biochemistry. Compared with the control, the FS+N treatment resulted in the increase of 56% photosynthetic efficiency, 9.6% nitrogen use efficiency, 60% nitrogen uptake, 80% soluble sugar, 59% starches, 48% biomass, and 29% grain yield of maize. In addition, the FS+N regime increased 47%, 42%, and 106% of soil organic carbon and available P and N content in comparison with the control. Maize grain and biomass yields were positively correlated with N uptake, photosynthesis, soil organic carbon, and soil available N and P contents. Conclusively, the use of wheat straw at 5000 kg ha^−1^, along with 172 kg N ha^−1^, is a promising option for building a sustainable wheat–maize cropping system to achieve optimal crop yield and improved plant and soil health in a semi-arid region of China.

## 1. Introduction

Land, water, and some other resources competition has rapidly increased with the enhancement in the demand for food [1,2]. Thus, about half of the word land area, including arid and semi-arid regions, and global food security problems can be fixed by improving the soil and plant health of these areas [3,4]. Increasing the yield per unit of grain is an effective measure to ensure food security. Low rainfall in the dryland is a direct threat to agricultural production; thus, it is very important to adopt a quick plan, especially improved management practices to overcome this problem and to enhance crop yield and production [5]. Further, the improper management of residues and fertilizer creates problems, such as water scarcity and the degradation of farmland, which are the key factors affecting worldwide agricultural production, especially in the semi-arid region of China. This has led to high vaporization rates and low soil moisture retention capacity, which intensify the imbalance between crop water demand and supply [6,7], leading to the decline of wheat production. Therefore, bettering soil health; improving soil structure, water storage, and moisture conservation capacity; and improving water use efficiency and crop yields have become important issues concerning agricultural practices. However, the endurance of higher and sustainable agricultural production depends on proper and suitable field practices [8], and must also be friendly for the ecological environment [9]. Thus, it is essential to address the impacts of field practices on the plant and soil. Crop straw is an important agricultural resource, which can also provide organic acids and neutralize the alkalinity of soil and increase soil organic carbon, which then improves soil quality. In addition, agriculture sustainability and water use efficiency are related to the use of straw mulch [10] and have, therefore, been widely used in crop cultivation [11,12]. Moreover, mulching has been shown to lead to significant changes in many biochemical processes in soil and, therefore, changes the soil microenvironment, thus increasing nitrogen availability [13] and accumulating more residual nitrogen in the soil [14]. The release of nitrogen from decomposing residues used as mulch is well synchronized with plant nitrogen uptake compared to inorganic nitrogen [15], which improves the availability of nitrogen and increases the nitrogen uptake efficiency and crop yield, while reducing nitrogen losses by leaching [16].

In northern China, excessive use of N fertilizers has led to elevated NO_3_-N concentrations in groundwater that often exceed drinking water standards [17]. However, this issue in semi-arid environments is characterized by an insufficient accumulation of soil surface residues due to the unnatural production of crop biomass. Therefore, the use of additional straw mulch for covering the soil surface can act as an agronomic tool to build up an adequate layer of residue over the soil surface, which controls nitrate leaching. Similarly, to control nitrate losses that result from the sole use of excessive fertilization, an integrated approach of synthetic fertilizer and mulching with straw residues is very important to reduce the downward movement of NO_3_-N into the soil profile and, subsequently, to groundwater, coupled with the maximization of N uptake efficiency and crop productivity. Straw mulching has received proper attention in areas that have more climatic perturbation and induced moisture and heat stresses. The greater photosynthetic efficiency due to straw mulching could be related to the greater moisture availability and enhanced microbial activity, improving mineralization and, thus, eventually increasing nutrients and plant performance. These enhanced nutrients, particularly N, which is considered a structural component of chlorophyll, improve the plant photosynthesis capability [18]. Using straw as mulch increases the crop growth rate, dry matter accumulation, and grain weight [19]. Previous studies have documented no changes in yield with mulching treatments [20]. However, long-term multi-location experiments using crop residues can make the cropping system sustainable [21].

Mulching enhanced the soil biotic activity of earthworms [22], other soil fauna, and the soil structure and quality [23]. Previous studies have mainly focused on soil temperature, moisture, bulk density, nutrient status, chemical properties, crop seedling emergence, diseases and pests, and grain yield and biomass in response to mulching techniques. However, limited information is available on the crop photosynthetic characteristics and their relationship with the maize grain/straw quality and quantity in the study area. Plant growth and development seem to be strongly affected by the agricultural measures of straw mulching in farmland systems. It is well known that chlorophyll is the basic material for plant photosynthesis, and its levels determine the photosynthetic rate up to a certain extent [24]. The improvement of the leaf photosynthetic characteristics has a significant impact on crop growth and development, dry matter accumulation, and final grain yield [25]. Most plant characteristics (physiology, growth, and yield) increase with increasing nitrogen, suggesting that nitrogen fertilizer is an important factor for improving the crop growth and yield of soybean [26] or winter wheat [27]. The increased N not only increases the plant parameters, but improves the soil moisture and temperature.

Different methods can be employed to return straw to the field, such as bang-up and direct plowing, covering the soil surface, composting, manuring, and biochar. The direct impact of these different practices are on soil functionalities for the improvement in soil health and crop production [28]. A field experiment determined that the soil NPK availability was significantly increased by straw and biochar amendments under the condition identical to carbon input [29]. However, to know the response of straw mulch in order to better utilize the recommended N fertilizer of the particular region has not been properly discussed. Therefore, with the abovementioned considerations, we studied the objectives of this research work, namely, that the effects of different levels of straw mulching, with or without inorganic N fertilizer, improve the physiology and growth traits; yield, plant, and soil functionalities; and water and nitrogen use efficiency of maize (*Zea mays* L.). Further, we also studied (i) to clarify the trends of maize chlorophyll content, photosynthetic rate, and yield with increasing straw mulch levels combined with inorganic N; (ii) to elucidate the correlations between the photosynthetic rate and yield; and (iii) to identify the changes in nitrogen uptake and its relationship with soil properties and maize yield.

## 2. Results

### 2.1. SPAD Value and Net Photosynthetic Rate

**Figure 1:**
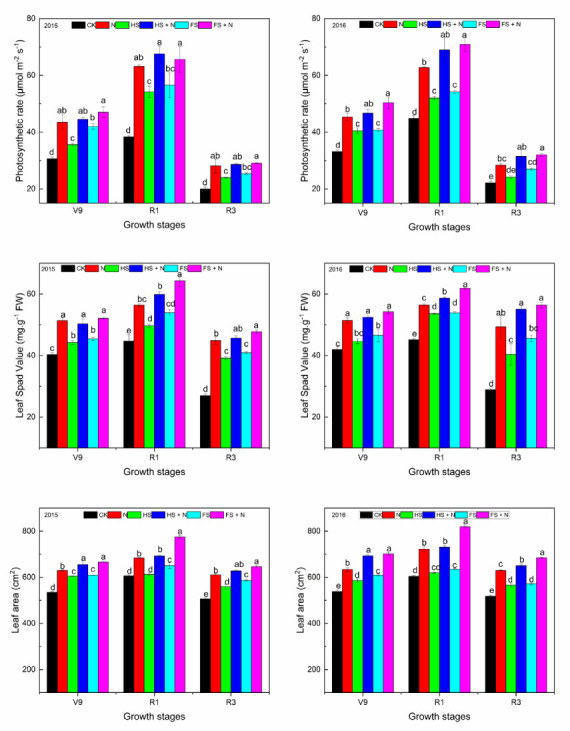
Variations in the net photosynthetic rate, leaf SPAD value, and flag leaf area across the different growth stages of maize under different mulching and nitrogen treatments (during 2015 and 2016). Note: V9—jointing, R1—silking, and R3—grain filling stage of maize. Statistical significance letter on each bar represents significant difference (*p* < 0.05) among different treatments in each year. During 2015 and 2016, the LSD values for the photosynthesis at V9, R1, and R3 stages were 4.7695, 8.4768, 3.5757 and 3.9655, 6.3573, 3.2693; Spad values were 2.9578, 4.4058, 2.3939 and 3.6682, 0.8966, 7.1031; and Leaf area values were 17.642, 19.434, 18.970 and 15.774, 20.770, 18.174.

The SPAD (Soil Plant Analysis Development) value was significantly affected by different treatments (Figure 1). Full straw mulch plus nitrogen fertilizer (FS+N) showed 46.6 and 48.8% higher SPAD values than the control in 2015 and 2016, respectively. There were no significant differences between full straw (FS), half straw (HS), nitrogen fertilizer (N), and half straw plus nitrogen fertilizer (HS+N) treatments, irrespective of the phenological phase. However, in 2015 and 2016, FS+N showed 29.4 and 29.3% increases in SPAD values at the V9 stage, 43.9 and 37.0% increases at the tasseling stage, and 76.9 and 95.4% increases in the R3 stage compared to no straw and no nitrogen fertilizer (CK) plots. These increases in SPAD values in response to mulching are reflected by the higher SPAD values compared to CK treatments.

The photosynthetic rate of leaves showed no significant differences among the FS+N, HS+N, and N treatments at the V9, R1, and R3 stages (Figure 1). Similarly, the differences were not significant between FS and HS treatments at the R1 and R3 stages (Figure 1). However, FS+N had a significantly higher photosynthetic rate than CK, HS, and FS at the V9, R1, and R3 stages. During 2015 and 2016, FS+N resulted in 53.6 and 52.1%, 71.2 and 58.4%, and 45.6 and 44.6% increases in photosynthetic rates at the V9, R1, and R3 stages, respectively, than CK. These results indicated that FS+N were more physiologically efficient than all other treatments, irrespective of the crop growth stage.

### 2.2. Flag Leaf Area and Crop Growth Rate

Significant differences among treatments were observed for the leaf area and crop growth rate (Figure 1; Table 1). The treatment FS+N had a significantly higher leaf area than all other treatments across the growth stages. However, no significant differences in the leaf area at the V9 stage were noted between the FS+N and HS+N treatments. It was further observed that FS+N had a significantly increased leaf area by 24.5 and 30.5% at the V9 stage, 27.9 and 35.6% at the R1 stage, and 27.6 and 32.2% at the R3 stage, compared with the CK regime. Similarly, the FS treatment had a 9.2 and 15.1%, 19.2 and 29.1%, and 10.2 and 19.6% higher leaf area at the V9, R1, and R3 stages, respectively, than CK (control). Significant differences among treatments in the crop growth rate were observed (Table 1). During 2015 and 2016, the FS+N, N, and FS treatments had 55 and 66.1 g d^−1^ m^−2^, 22.2 and 29.1 g d^−1^ m^−2^, and 11.2 and 15.8 g d^−1^ m^−2^ higher crop growth rates, respectively, than CK. Similarly, a mean difference of 47.1 g d^−1^ m^−2^ in the crop growth rate between FS+N and FS treatments was observed (Table 1).

### 2.3. Maize Production and WUE

**Figure 2:**
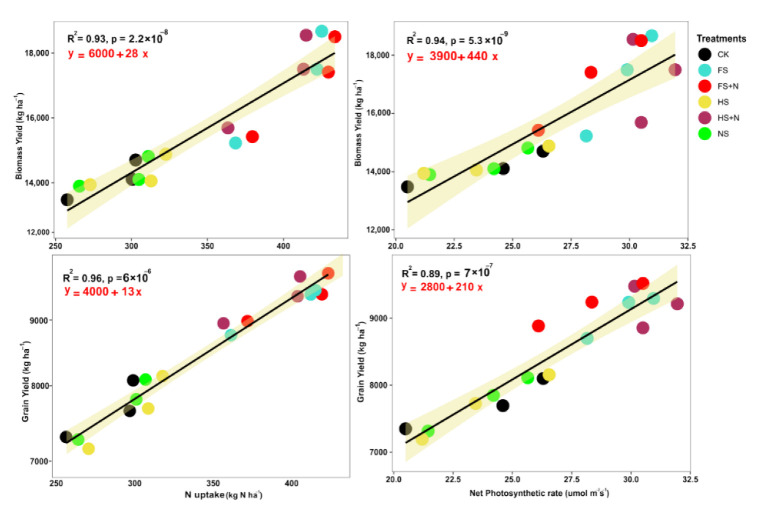
Linear regression of N-uptake and photosynthetic rate with biomass and grain yield of maize pooled over years.

The yield contributing components, i.e., grains ear^−1^ and thousand-grain weights, showed significant differences for different treatments (Table 1). Over two years’ averages, the wheat straw mulching at the full rate (with or without N) improved the grain yield and yield components of maize. More specifically, an increase of 18.7, 12.4, and 5.5% in plant height; 24.6, 33.9, and 16.6% in grains per cob; and 30.6, 16.9, and 11.0% in thousand-grain weight were recorded for FS+N, N, and FS treatments, respectively, when compared with CK. However, no statistical differences in maize plant height were observed in response to various treatments (i.e., FS+N and HS+N, FS and HS). The mulching treatments had showed significant differences for biomass yield and grain yield (*p* ≤ 0.05), as shown in Table 1. The two-year average, more grain yield (9433 kg ha^−1^), and biomass yield (18,572 kg ha^−1^) were recorded for the FS+N treatment as compared to other treatments, with the minimum recorded in the CK treatment. The water use efficiency average over the two years was higher in FS+N (19.6 kg ha^−1^ mm^−1^) and more under the FS+N treatment than the rest of the treatments (Table 1). Specifically, the sole wheat straw residue as mulching had a higher WUE than CK or N, but lower than combined wheat straw mulching and N fertilization. This indicates the positive effects of fertilizer addition in improving the WUE as a result of increased plant performance. The plant photosynthesis was positively correlated with biological yield (R^2^ = 0.94) and grain yield (R^2^ = 0.89), with a linear trend (Figure 2). 

### 2.4. Maize Nitrogen Content and Uptake

The application of mulch, with or without N, showed significant effects on the tissue nitrogen content, NUE, and its uptake by maize over two years (Table 2). During 2015 and 2016, the treatment FS+N resulted in maximum straw nitrogen (9.3 and 13.1 g kg^−1^) and grain nitrogen contents (26.3 and 58.1 g kg^−1^) compared to the control plots. The two-year average integration of N, with half or full straw application, showed no differences in the grain total nitrogen but did improve the NUE significantly by 32.5% with FS+N regimes as compared to HS+N (31.2%) or N (30.1%). Furthermore, straw mulching with inorganic N had strong effects on maize N uptake (Table 2). Over the two-year study, the regime FS+N showed higher N uptake compared to that of the control (CK). Furthermore, all treatments had significant increases in N uptake over the CK treatment. The N uptake showed a positive and significant relationship (Figure 2) with grain yield (R^2^ = 0.96) and biomass yield (R^2^ = 0.93).

### 2.5. Soluble Sugar and Starch Content

**Figure 3:**
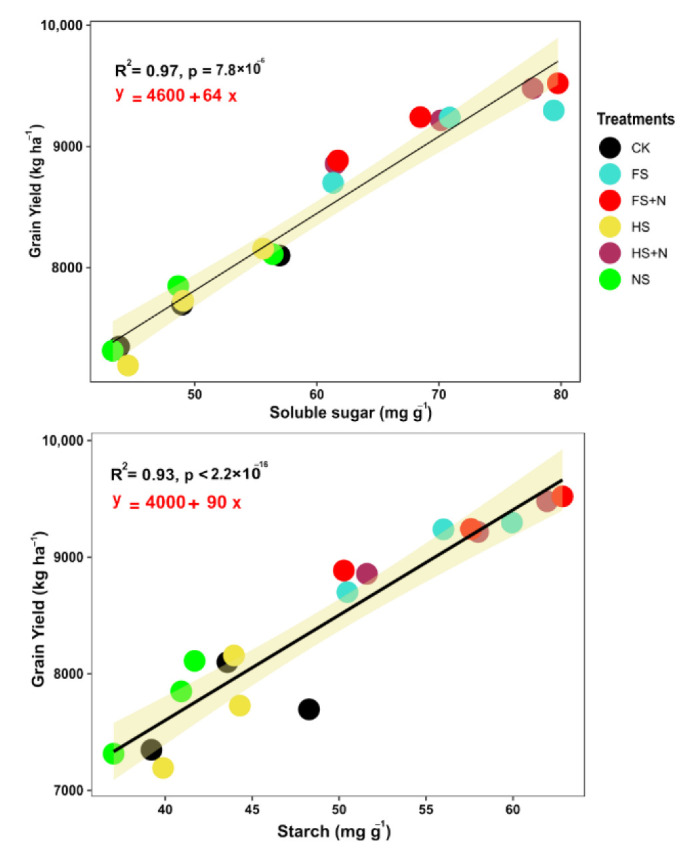
Linear regression of soluble sugar and starch with grain yield of maize pooled over years.

The treatments had significant effects on the soluble sugar and starch both in maize straw and grain (Table 3). The FS+N treatment had 20.5 and 22.1 mg g^−1^ higher soluble sugar content than CK in maize straw. The addition of N to either the half or full rate of mulching treatments resulted in a higher soluble sugar content in stover compared to mulch treatments without N. Similarly, the soluble sugar in grain was 11.9 and 14.0 mg g^−1^ higher in the FS+N treatment than in CK. Mulching treatments, used at half or full rates, had lower soluble sugar contents in maize grain compared to the soluble sugar measured in treatments with mixed mulch + N. Generally, the starch contents, both in the straw and grain of maize, increased with the addition of N to the mulching treatments compared to sole mulching treatments. Specifically, the maximum starch contents in straw (12.3 and 6.9 mg g^−1^) and grains (16.4 and 10.2 mg g^−1^) were observed for the FS+N treatment compared to the minimum starch contents recorded in control plots. It was further observed that FS+N treatments increased the starch content by 4.4 times in stover, but 1.4 times in grains compared to the control treatments. A positive and significant relationship of soluble sugar and starch was observed for the grain yield of maize (Figure 3).

### 2.6. Changes in Soil Organic Carbon and Soil N, P, and Moisture Contents

**Figure 4:**
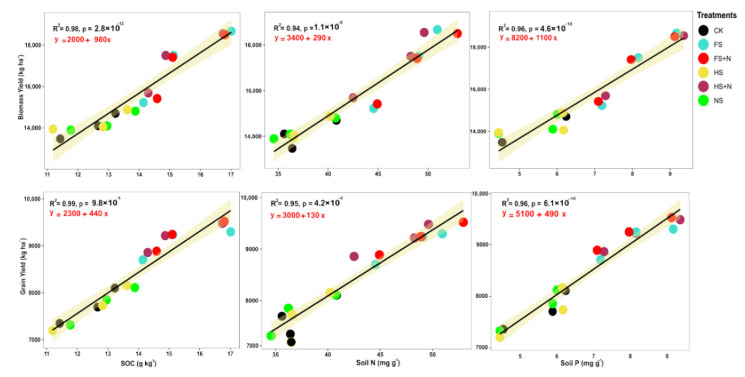
Linear regression of SOC and soil N and P with biomass and grain yield of maize pooled over years.

**Figure 5:**
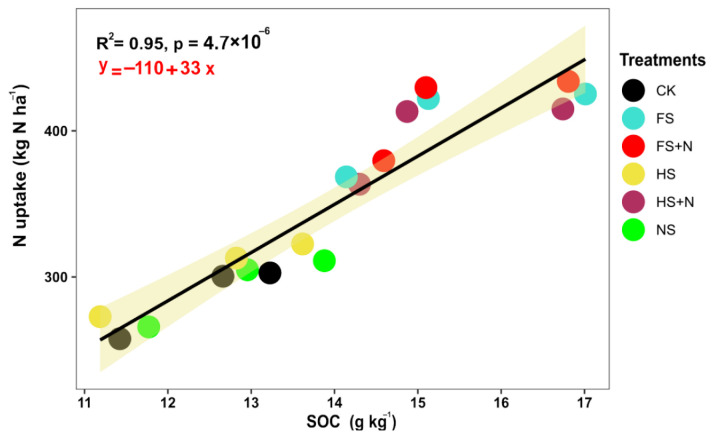
Linear regression of soil organic carbon with N uptake of maize pooled over years.

**Figure 6:**
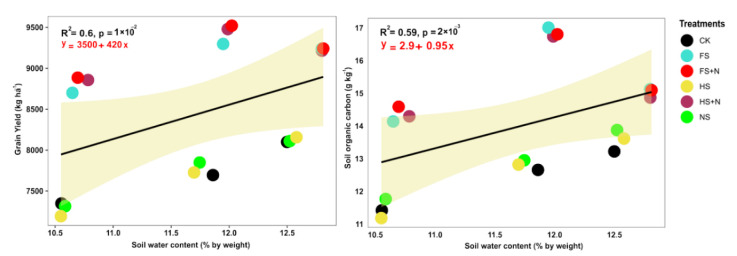
The relationship of soil moisture with soil organic carbon and grain yield of maize pooled over years.

Changes in soil organic carbon (SOC) and the N, P, and moisture contents after the harvest of maize are shown in Table 4. The treatments showed significant effects on SOC and the available N and P and water contents. The treatments containing straw mulch with inorganic N increased the SOC, the soil N and P, and the soil water content (SWC), unlike the mulch treatments without N. The highest content of SOC, soil N and P, and SWC was recorded using full straw mulch in combination with inorganic N (FS+N). Likewise, the control plots had the minimum SOC, soil N and P, and SWC contents. The grain yield and biomass yield showed a linear positive correlation with SOC and soil N and P (Figure 4). The relationships among soil C, N, and P were significantly positively correlated with biomass and grain yield (Figure 4). Additionally, SOC was positive and linearly significant with N uptake (Figure 5). In addition, soil water content was significantly positively correlated with SOC (R^2^ = 0.59), and grain yield (R^2^ = 0.6) with a linear trend (Figure 6). 

### 2.7. Relationship between Grain Yield and WUE

**Figure 7:**
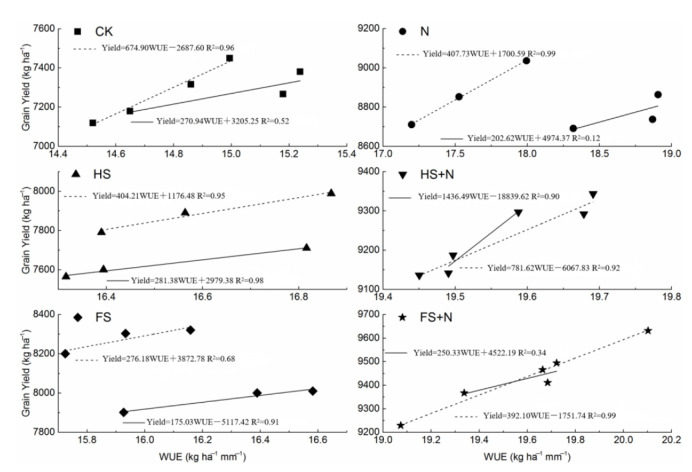
The relationship between grain yield with water use efficiency in different treatments during 2015 and 2016. (Note: solid line represents data for 2015; dash line represents data for 2016).

The linear fit regression analysis for grain yield and WUE under different treatments is presented in Figure 7. The regression analysis revealed that the change in WUE had positively affected the grain yield with respect to different treatments during 2015 and 2016. The results indicated that the relationship between WUE and grain yield was more effective during 2016, when inorganic N was used along with full straw mulch (FS+N). More specifically, no changes in the slope were observed during 2015 or 2016 when HS or FS were used; however, the slope increased in 2016 over 2015 when N was augmented with HS or FS.

## 3. Discussion

Mulching practices are carried out for soil moisture conservation and its associated benefits, particularly in semi-arid regions, such as China. This increase in soil moisture can occur by two mechanisms: (a) improving the soil organic matter content, which increases the aggregate stability and porosity [30]; (b) developing physical barriers at the surface, which reduces the evaporation and soil surface runoff [31]. In our studies, we used wheat straw as mulching material, which can provide organic matter to soil upon decomposition and can also act as a physical barrier until it has been decomposed. Wheat straw has a significant amount of carbon content, though with a quite wide C: N ratio. Therefore, wheat straw mulch with the addition of N fertilizer was considered to be a useful tool to better utilize the recommended inorganic fertilizer compared with that of sole use of inorganic fertilizer, and also enhances the production of the following maize crop and the associated benefits to soil health.

### 3.1. Photosynthetic Rate and Harvest Measurements

Mulching with only straw or N + straw increased the plant’s photosynthetic capability compared to control plots. The straw mulch covers the soil, which ensures enough living and/or residual biomass, with improved soil moisture contents. Straw mulching has received proper attention in areas experiencing climatic perturbations and induced moisture and heat stresses. In the current study, the SPAD value and photosynthetic rate, measured at the V9, R1, and R3 stages, were found to be higher in plants grown in FS+N compared with no-mulch plots (CK). The increased photosynthetic efficiency in the FS+N treatments could be related to the greater moisture availability and make soil nutrients available for plants. Combining straw mulching with organic fertilizer improved soil physical and chemical properties, promoted root growth and nutrient absorption, and increased the generation of root secretions, which enhanced the soil microbial biomass quantity and soil microbial activity, which subsequently improved mineralization and nutrient uptake [32,33]. The improved mineralization and nutrient uptake likely enhanced the nutrient content, particularly N, which, as a structural component of chlorophyll, improved the plant photosynthesis capability [34]. Water stress decreases crop growth and development and, consequently, lowers the optimum stover N uptake required by the plant and may be an impediment for achieving high yield, and it also reduces the plant’s accessibility for N uptake [35]. The conserved optimum moisture and improved contents/chlorophyll in the FS+N treatments might have increased the LA, crop growth rate, yield attributes, and yield. Similarly, it was most probably associated with better water and N availability, which might have helped better crop growth and N uptake in the growing seasons, contributing to a higher maize grain yield [36]. However, when we added a basic dose of N, the mulching treatments showed a significant response to most of the studied parameters. Long-term multi-location experiments suggested that using crop residues can make cropping sustainable [37]. Similarly, using straw as a mulch increased the crop growth rate, dry matter accumulation, and grain weight [38]. In our studies, the significant positive linear relationship of biomass and the grain yield with the photosynthetic rate could be associated with the strong impact of mulching treatments rather than other factors, as suggested by Guo et al. [39]. Similarly, leaf area is the basic building block for the increased photosynthetic rate, which increases dry matter production via increased photo-assimilate formation. Straw mulching improves soil water content, increases flag leaf photosynthetic parameters, and maintains the yield of winter wheat with different irrigation amounts [40], and this phenomenon is supported by the relationship of flag leaf photosynthesis and the crop growth rate, which are interrelated. Thus, we can consider leaf photosynthesis as a key factor for enhancing biomass yield.

### 3.2. N-Uptake and Yield

The uptake of nitrogen can be considered a physiological index to measure the efficiency of plants for nitrogen. The higher the N uptake, the more N is transferred from the soil into plants, and thus, the plants are said to be more physiologically efficient. Our results indicated that with the increase in straw mulch, along with nitrogen fertilizer, the N uptake and grain yield significantly increased compared to CK. The probable reasons for the increased N uptake could be associated with the increased availability of directly added N. The decreased soil surface temperature [41,42] and lower water losses in mulched plots have been reported to increase the grain yield and nutrient uptake in soybean crops [43]. Similarly, the increased decomposition of crop residues led to an increase in the release of nitrogen, which corresponded with plant N uptake, irrespective of the fertilizer source of N, and resulted in decreased N losses and, hence, increased the N uptake and crop yield [44,45]. These arguments support the trend of our data, indicating positive and linear relationships of N uptake with the grain and the biological yields of maize. The integration of wheat straw mulching with N is believed to improve the crop growth, which is clearly observed from our data. The increased slope for FS+N and HS+N principally in the following year than sole FS or HS indicates the increased N availability impact on crop production. These results hold true when comparing the WUE and crop growth: the crop growth was improved with N fertilization, along with wheat straw residue.

### 3.3. Soluble Sugar and Starch Effects on Grain Yield

Soluble sugar is considered an important media for storing and transferring energy for C metabolism. The positive linear relationship of soluble sugar/starch with the grain yield indicated that more energy was provided for improving the grain yield. Our results showed that the mulching with inorganic N significantly enhanced the content of soluble sugar and starch in the straw and grains of the maize crop at the harvest stage (Table 3). This increase in soluble sugar is associated with vigorous plant growth; increased photo-assimilate production as a result of a higher leaf area resulted in increased soluble sugar/starches in both straw and grains of maize crops. The increases in the starch content also depended on genetic and environmental factors [46]. However, in the current study, the same maize genotype was evaluated under the same field conditions; therefore, the difference is apparently more related to management practices, rather than the genetic x environment interaction. The added straw as mulch improved the soil properties, thereby positively affecting the accumulation of dry matter, starch content, and the final yield and quality of crops [47]

### 3.4. Changes in Soil Fertility in Response to Mulching

Use of an organic residue as mulch is believed to improve both the biological and chemical properties of soil [48]. Our results showed significant positive changes in soil organic carbon and available N and P with the application of wheat straw as mulch. The soil fertility in terms of SOC and available P and N was higher under the FS+N treatment compared with the rest of the treatments. The added straw acted as a major source of nutrients and soil organic matter, which increase the soil quality, and the microbial communities were also influenced, thus boosting the production of labile C and N components and accelerating the C and N cycle in maize fields [49,50]. This increased soil quality might have increased the soil biological properties and, hence, improved the microbial activities, thereby increasing the soil available N as a result of the higher net mineralization of added straw mulch [51]. Likewise, the improved soil physicochemical properties [52] with the addition of a crop residue played a model role in nutrient cycling and improving soil organic matter dynamics [53].

The high SOC is believed to lead to better soil health and fertility. Therefore, improvements in the SOC contents are a prerequisite for increasing the nutrient use efficiency, crop growth, yield, and soil quality [54]. In the current study, aboveground crop responses in terms of enhanced biological and grain yields were correlated positively with belowground increases in SOC and available N and P as a result of added straw mulching. This increased SOC from wheat straw is an important mechanism, leading to increased mineralization and, hence, increased crop yields and nutrient uptake and positive changes in the soil physical and biochemical properties. Previous studies have documented positive correlations between SOC and crop biological yields [55], though with little or almost negligible convincing proof of the relationship between SOC and the crop yield. Therefore, the significant correlations of crop productivity and yields with SOC in this study should also be interpreted thoughtfully, either based on increased nutrient availability or other soil properties.

## 4. Materials and Methods

### 4.1. Experimental Site 

**Figure 8:**
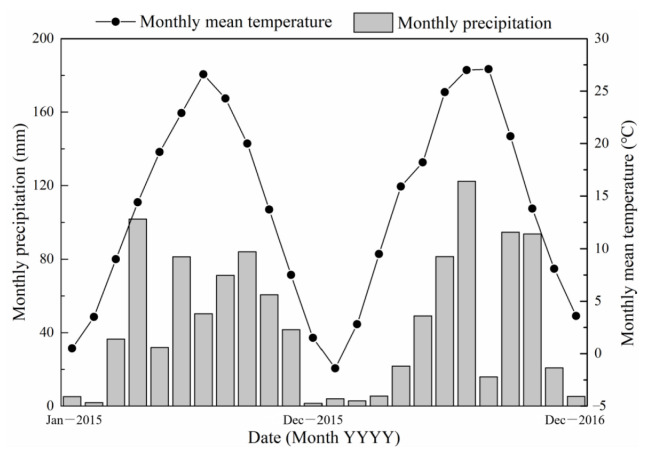
Monthly precipitation and temperature during 2015 and 2016.

**Figure 9:**
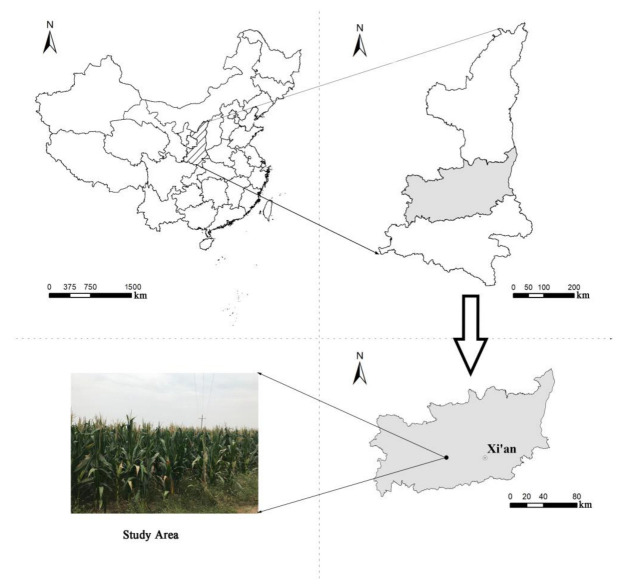
A map of geographical location for the experimental area in China.

The experimental site (34°12′ N and 108°07′ E) is situated at Northwest A & F University, China, Yangling, Shaanxi Province. The area is 520 m above sea level. The mean annual temperature and precipitation are 12.9 °C and 660 mm, respectively, and the precipitation is mainly concentrated from July to September. The soil is classified as Lou soil (anthrosol), and the soil texture is silty clay loam, with a pH of 8.3, bulk density 1.53 g cm^−1^, saturated soil water content 44%, field capacity 22.4%, EC 170 µS cm^−1^, available N 26.5 mg kg^−1^, available P 10.2 mg kg^−1^, available K 132 mg kg^−1^, soil organic carbon 11.2 g kg^−1^, and soil water contents 8.7% at 0.2 m depth of soil. Soil nutrients were measured following the methods of Bao [38]. The precipitation and temperature data are presented in Figure 8, while a map of the geographic location for the experimental area is presented in Figure 9.

### 4.2. Experimental Design

The experimental design was a randomized complete block with six treatments and three replicates, having a total of 18 plots, and each plot had a size of 8 × 8.25 m. This is a wheat–maize cultivated area, and each year after the harvest of wheat, the remaining wheat straw was used for mulch practice. Further, we have selected the remaining wheat straw from each plot as full straw (5000 kg ha^−1^) and then selected half straw (2500 kg ha^−1^) on the basis of full straw, while nitrogen doze was considered as the recommended doze of the particular region. Therefore, during 2015 and 2016, treatments were arranged in an order such as CK (control), N (no wheat straw mulching with 172 kg N ha^−1^), HS (half wheat straw mulching at the rate of 2500 kg ha^−1^), HS+N (half wheat straw mulching at a rate of 2500 kg ha^−1^, with 172 kg N ha^−1^), FS (full wheat straw mulching at a rate of 5000 kg ha^−1^), and FS+N (full wheat straw mulching at a rate of 5000 kg ha^−1^, with 172 kg N ha^−1^) to quantify their effect on the soil moisture conservation, water use efficiency, soil fertility and maize productivity, photosynthesis, and N uptake.

During wheat harvesting, the wheat residue (containing 0.5% N) was chopped into 3–5 cm pieces and was maintained as mulch for the next maize crop consecutively for two years (2015 and 2016). Maize sowing was conducted each year in June 2015 and 2016. Urea was applied as the source of nitrogen every year at the V9 stage. The summer maize (cultivar Luo dan No. 9) was planted at a rate of 60 kg ha^−1^ on 15 June 2015 and 16 June 2016 at a constant depth (5 cm), using a no till planter machine while maintaining 60,333 plants ha^−1^. The row spacing was 75 cm, and plant spacing was 25 cm. Manual weeding was conducted as required during the field experiment each year. Each year, irrigation of 120 mm was provided at the V9 stage of the maize. The surface irrigation of 120 mm of water is a general practice in the studied area. The experimental field was not plowed before sowing the maize crop.

### 4.3. Observations and Measurement

#### 4.3.1. SPAD Readings

The estimation of chlorophyll concentration in leaves was obtained with the SPAD (soil plant analysis development) 502 plus a portable chlorophyll meter (KonicaMinolta, Inc., Tokyo, Japan). Several measurements were taken on the mid-section of 10 fully expanded leaves per plot at the V9, R1, and R3 growth stages of maize. SPAD measurements were made on the flag leaf if present or otherwise with the greenish leaves to avoid the variation in SPAD values in the youngest leaves (before anthesis) and older leaves (after anthesis) [56].

#### 4.3.2. Net Photosynthetic Rate

A portable photosynthesis system (LI-6400, LI-COR, Lincoln, NE, USA) was used to measure the flag leaf photosynthetic rate at the V9, R1, and R3 stages of maize. Maize ear leaves were selected for photosynthesis measurements, and the measurements were made at five points per leaf between 10:00 and 11:30 h local time.

#### 4.3.3. Flag Leaf Area and Crop Growth Rate

Five plants from an area of 0.75 m^2^ were harvested from each experimental unit at the V9, R1, and R3 stages of maize. Flag leaves were removed from all these plants, and the leaf area of all these flag leaves was measured using a leaf area meter (DT Area Meter, Model MK2, Delta T Devices, Cambridge, UK). The dry weights of all five plants were measured, and the crop growth rate (CGR) was calculated using the formula proposed by [57].

### 4.4. Harvest Measurements

At maturity, four central rows were harvested, sun dried, and weighed to record data on the biological yield of maize. The ears of maize were shelled and weighed to record the grain yield. Thousand-grain weights (TGWs) were counted on a Contador (Pfeuffer, Germany) seed counter, and the weight was recorded with the help of a balance. The grains per ear were calculated by dividing the grain weight of ears by the mean grain weight (i.e., TGW/1000). The water use efficiency (WUE) was calculated by dividing the grain yield by the evapotranspiration (ET) of the growing season [58] using the following equation.

(1) WUE=Y/ET

where Y is the grain yield (kg ha^−1^), and ET is the total evapotranspiration (mm) over the growing season and was calculated by the following equation.

(2) ET=SWS1−SWS2+P+I

where ET is the evapotranspiration (mm), SWS1 is the soil water storage at sowing, SWS2 is the soil water storage at harvesting in the 0–20 cm soil profile (mm), (P) is the precipitation (mm), and (I) is the irrigation amount (mm).

#### 4.4.1. Plant N Analysis and Nitrogen Indices

The concentrations of N in the straw and grain of maize were measured in each plot, using the Dumas method on the hand-harvested samples. The N-uptake and nitrogen use efficiency (NUE) were calculated by the formula of Wolf [59].

(3) $$N\;\mathrm{uptake}\;\left(\mathrm{kg}\;{ha}^{-1}\right)=\frac{\mathrm{Harvest}\;N\;\mathrm{content}\;\left(g\;{kg}^{-1}\right)\;\times \;\mathrm{grain}\;\mathrm{yield}\;\left(\mathrm{kg}\;{ha}^{-1}\right)}{1000}$$

(4) $$\mathrm{NUE}\;(\%)=\frac{\mathrm{Accumulated}\;N\;(\mathrm{kg}\;{ha}^{-1}\;\mathrm{in}\;\mathrm{fertilized}\;\mathrm{plot}\;-\;\mathrm{control}\;\mathrm{plot})}{\mathrm{Total}\;N\;\mathrm{applied}\;(\mathrm{kg}\;{ha}^{-1})}\times \;100$$

#### 4.4.2. Plant Soluble Sugar and Starch

The soluble sugar and starch contents, both in the straw and grain of maize, were determined using hand-harvested samples. The soluble sugar and starch contents were measured in straw and grain following the method of Zhao et al. [54].

#### 4.4.3. Soil Sampling and Analysis

Five random soil samples were collected annually from each plot during September of each year, after maize harvest, from a soil depth of 0.2 m. In each plot, at five different points, five subsoil samples were collected and mixed by quartering to make a composite sample and were then divided into two parts. One part was dried at room temperature and was used for soil nutrient analysis, and the second part was used for soil moisture determination. The oven-drying method was adopted to measure the soil water content (% by weight) by using a temperature of 105 °C, and the samples were removed from the oven after 12 h. The available nitrogen was determined using 1.0 M KCl extraction, following the cadmium reduction method [46]. The available phosphorus was extracted by an NaHCO_3_ Ultraviolet Spectrometer Subsystem (UVS) [60]. Soil organic carbon (SOC) was determined following the K_2_Cr_2_O_7_–H_2_SO_4_ digestion method [61].

### 4.5. Statistical Analysis

For each variable, the mean values were calculated, and for the comparison of different treatments, a one-factor an analysis of variance (ANOVA) was used. The means were compared by the Least Significant Difference test at *p*  ≤ 0.05. The statistical analyses were performed using SPSS 20.0. A linear regression analysis was tested for photosynthesis, N uptake, SOC, soil N and P with biomass and grain yield, SOC with N uptake, and WUE, as well as the soluble sugar and starch with the grain yield of maize. Pearson’s correlation was conducted for soil moisture with the soil organic carbon and grain yield of maize.

## 5. Conclusions

Straw mulching has received proper attention in areas that have more climatic disturbance and induced moisture and heat stresses. The results of this study indicated that straw mulch combined with inorganic N fertilizer has a significant positive effect on the soil functionalities via enhancing the physiology, growth, yield, and N-uptake of maize. Therefore, the addition of wheat straw as a mulch material, along with commercial N (FS+N), showed promising improvements in the physiological efficiency, plant growth, and nutrient availability. The increased soil nutrients significantly correlated with the crop, including physiological responses and a higher number of grains cob^−1^ and an increased crop growth rate, photosynthesis rate, soluble sugars/starches, and crop yields. We also postulate that mulching with commercial fertilization increased the soluble sugar in the semi-arid region and, thus, improved WUE and developed drought resistance, with a concomitant increase in crop production. Under our experimental conditions, in which maize was very often the main crop following wheat cropping, 5000 kg ha^−1^ of wheat straw added as mulch with 172 kg N ha^−1^ (FS+N) was sufficient to produce a significantly higher grain yield, with the increase in nitrogen and water use efficiency, and qualitative traits of maize, on a sustainable basis, as evidenced by the improved soil organic carbon and available P and N contents. Isotopic quantification of the relative contributions of wheat straw and fertilizer to the various plant and soil pools is recommended for understating that the mechanistic approach to such a phenomenon is the best option for the better utilization of the recommended inorganic fertilizer of the particular region.

## Figures and Tables

**Table 1:** Maize crop growth and production in response to straw mulching and nitrogen treatments.

Treatments	Plant Height (cm)	Grains ear^−1^	TGW (g)	CGR (g d^−1^ m^−2^)	Biomass Yield (kg ha^−1^)	Grain Yield (kg ha^−1^)	WUE (kg ha^−1^ mm^−1^)
	2015						
CK	201 c	490 c	304.3 f	15.0 e	13,657 f	7275 f	15.0 d
N	225 b	627 a	363.5 c	37.2 c	15,553 c	8963 c	18.7 b
HS	210 c	563 b	332.5 e	19.2 de	14,000 e	7625 e	16.3 c
HS+N	229 b	590 ab	368.0 b	51.1 b	16,010 b	9208 b	19.5 a
FS	211 c	546 b	335.2 d	26.2 d	14,627 d	7970 d	16.5 c
FS+N	237 a	582 ab	399.7 a	70.0 a	16,927 a	9424 a	19.6 a
LSD	10.0	45.51	2.5	7.1	265.8	117.3	0.326
	2016						
CK	205 d	478 d	313.3 e	12.6 f	13,883 d	7295 e	14.8 e
N	230 b	669 a	366.2 c	41.7 c	15,337 c	8866 b	17.6 b
HS	215 c	605 bc	341.5 d	20.7 e	14,173 d	7889 d	15.9 d
HS+N	234 ab	632 ab	379.7 b	59.4 b	18,930 b	9257 a	19.6 a
FS	216 c	582 c	347.8 d	28.4 d	14,967 c	8275 c	16.6 c
FS+N	243 a	624 b	409.2 a	78.7 a	20,217 a	9442 a	19.6 a
LSD	9.33	40.69	7.22	5.95	572.9	266.8	0.629

Note: Thousand-grain weight (TGW), Crop growth rate (CGR), Water use efficiency (WUE). CK (no amendments); N (172 kg N ha^−1^); HS (half straw mulching at a rate of 2500 kg ha^−1^ of wheat straw); HS+N (half straw mulching at a rate of 2500 kg ha^−1^ of wheat straw with 172 kg N ha^−1^); FS (full straw at a rate of 5000 kg ha^−1^ of wheat straw); FS+N (full straw at a rate of 5000 kg ha^−1^ of wheat straw with 172 kg N ha^−1^). Values within a column for the same year followed by different letters are significantly different (*p* < 0.05).

**Table 2:** Maize N contents, N uptake, and nitrogen use efficiency (NUE) in response to straw mulching and nitrogen treatments.

	Straw Mulching	Stover N (g kg^−1^)	Grain N (g kg^−1^)	N Uptake (kg ha^−1^)	NUE (%)
2015	CK	17.3 ± 0.07 f	21.3 ± 0.15 e	281 ± 3.8 e	
	N	20.1 ± 0.17 c	22.5 ± 0.06 c	374 ± 4.1 c	27.8 ± 0.02 c
	HS	17.6 ± 0.09 e	21.7 ± 0.13 d	300 ± 3.4 d	
	HS+N	21.0 ± 0.11 b	23.1 ± 0.20 b	407 ± 4.1 b	29.8 ± 0.27 b
	FS	17.8 ± 0.04 d	21.5 ± 0.10 de	299 ± 3.7 d	
	FS+N	21.8 ± 0.19 a	23.3 ± 0.24 a	425 ± 5.7 a	32.5 ± 0.84 a
	LSD	0.21	0.25	2.90	1.70
2016	CK	13.7 ± 0.01 f	20.6 ± 0.18 e	250 ± 4.9 f	
	N	18.8 ± 0.15 d	22.6 ± 0.02 b	367 ± 5.4 c	27.9 ± 0.69 b
	HS	18.4 ± 0.14 e	21.2 ± 0.09 d	313 ± 4.0 e	
	HS+N	24.0 ± 0.21 a	23.2 ± 0.10 a	436 ± 5.8 a	33.3 ± 0.43 a
	FS	20.4 ± 0.18 c	22.1 ± 0.28 c	325 ± 7.8 d	
	FS+N	21.6 ± 0.19 b	23.3 ± 0.10 a	424 ± 6.1 b	26.6 ± 1.21 b
	LSD	0.40	0.30	5.70	3.9

Note: CK (no amendments); N (172 kg N ha^−1^); HS (half straw mulching at a rate of 2500 kg ha^−1^ of wheat straw); HS+N (half straw mulching at a rate of 2500 kg ha^−1^ of wheat straw with 172 kg N ha^−1^); FS (full straw at a rate of 5000 kg ha^−1^ of wheat straw); FS+N (full straw at a rate of 5000 kg ha^−1^ of wheat straw with 172 kg N ha^−1^). Values within a column for the same year followed by different letters are significantly different (*p* < 0.05).

**Table 3:** Soluble sugar and starch content (mg g^−1^ dry matter) of maize is affected by straw mulching and nitrogen treatments.

	Straw Mulching	Soluble Sugar (mg g^−1^)		Starch (mg g^−1^)	
		Stover	Grain	Stover	Grain
2015	CK	16.1 ± 0.21 f	28.0 ± 0.01 e	2.33 ± 0.24 d	37.2 ± 0.30 d
	N	26.2 ± 0.40 c	33.1 ± 0.63 c	5.50 ± 0.24 c	46.1 ± 0.40 b
	HS	19.2 ± 0.10 e	28.4 ± 0.44 e	4.72 ± 0.17 c	39.5 ± 0.70 cd
	HS+N	31.4 ± 0.80 b	35.5 ± 0.23 b	7.71 ± 0.27 b	47.9 ± 0.18 ab
	FS	24.3 ± 0.60 d	30.9 ± 0.24 d	5.86 ± 0.85 c	40.4 ± 1.03 c
	FS+N	36.6 ± 0.03 a	39.9 ± 0.23 a	9.25 ± 0.24 a	49.5 ± 1.23 a
	LSD	1.25	1.20	1.34	2.26
2016	CK	15.3 ± 0.67 f	28.4 ± 0.35 e	2.65 ± 0.24 d	35.2 ± 1.78 d
	N	29.4 ± 0.60 c	34.3 ± 0.33 c	5.27 ± 0.52 c	44.6 ± 1.63 b
	HS	20.6 ± 0.18 e	29.6 ± 0.20 e	4.47 ± 0.06 c	40.3 ± 0.91 c
	HS+N	33.2 ± 0.62 b	39.5 ± 0.73 b	7.72 ± 0.37 b	51.0 ± 1.04 a
	FS	25.9 ± 0.19 d	31.5 ± 0.26 d	2.28 ± 0.13 d	37.6 ± 0.66 cd
	FS+N	38.9 ± 0.47 a	42.4 ± 0.83 a	12.8 ± 0.59 a	51.6 ± 0.47 a
	LSD	1.56	1.61	1.21	2.91

Note: CK (no amendments); N (172 kg N ha^−1^); HS (half straw mulching at a rate of 2500 kg ha^−1^ of wheat straw); HS+N (half straw mulching at a rate of 2500 kg ha^−1^ of wheat straw with 172 kg N ha^−1^); FS (full straw at a rate of 5000 kg ha^−1^ of wheat straw); FS+N (full straw at a rate of 5000 kg ha^−1^ of wheat straw with 172 kg N ha^−1^). Values within a column for the same year followed by different letters are significantly different (*p* < 0.05).

**Table 4:** Changes in soil properties in response to straw mulching and nitrogen.

	Treatments	Soil Organic Carbon (g kg^−1^)	Available Nitrogen (mg kg^−1^)	Available Phosphorus (mg kg^−1^)	Soil Moisture Content (%)
2015	CK	12.9 ± 0.25 d	30.9 ± 0.66 f	3.43 ± 0.03 e	6.87 ± 0.14 f
	N	14.6 ± 0.21 b	40.0 ± 0.09 c	5.29 ± 0.11 c	7.87 ± 0.14 c
	HS	13.4 ± 0.16 c	34.6 ± 0.15 e	4.60 ± 0.11 d	7.29 ± 0.10 d
	HS+N	14.9 ± 0.08 b	41.3 ± 0.30 b	6.36 ± 0.08 b	8.24 ± 0.12 b
	FS	13.5 ± 0.27 c	37.4 ± 0.42 d	5.23 ± 0.06 c	7.13 ± 0.11 e
	FS+N	15.8 ± 0.07 a	43.8 ± 0.12 a	7.84 ± 0.14 a	8.59 ± 0.11 a
	LSD	0.17	0.54	0.14	0.07
2016	CK	10.0 ± 0.21 e	40.7 ± 0.72 cd	5.53 ± 0.08 f	12.0 ± 0.11 e
	N	14.1 ± 0.33 c	48.0 ± 1.55 b	9.90 ± 0.01 c	13.0 ± 0.10 d
	HS	12.2 ± 0.15 d	37.7 ± 0.71 d	7.36 ± 0.08 d	13.0 ± 0.11 d
	HS+N	15.2 ± 0.08 b	56.2 ± 0.32 a	9.82 ± 0.14 b	13.2 ± 0.11 b
	FS	13.7 ± 0.14 c	43.8 ± 0.15 c	7.03 ± 0.08 e	13.1 ± 0.11 c
	FS+N	17.9 ± 0.11 a	58.5 ± 1.92 a	10.6 ± 0.01 a	13.5 ± 0.15 a
	LSD	0.29	1.38	0.12	0.07

Note: CK (no amendments); N (172 kg N ha^−1^); HS (half straw mulching at a rate of 2500 kg ha^−1^ of wheat straw); HS+N (half straw mulching at a rate of 2500 kg ha^−1^ of wheat straw with 172 kg N ha^−1^); FS (full straw at a rate of 5000 kg ha^−1^ of wheat straw); FS+N (full straw at a rate of 5000 kg ha^−1^ of wheat straw with 172 kg N ha^−1^). Values within a column for the same year followed by different letters are significantly different (*p* < 0.05).

## Data Availability

Data will be made available on demand.
